# First *in vivo* fluorine-19 magnetic resonance imaging of the multiple sclerosis drug siponimod

**DOI:** 10.7150/thno.77041

**Published:** 2023-02-05

**Authors:** Ludger Starke, Jason M. Millward, Christian Prinz, Fatima Sherazi, Helmar Waiczies, Christoph Lippert, Marc Nazaré, Friedemann Paul, Thoralf Niendorf, Sonia Waiczies

**Affiliations:** 1 Max-Delbrück-Center for Molecular Medicine in the Helmholtz Association (MDC), Berlin Ultrahigh Field Facility, Berlin, Germany; 2 Hasso Plattner Institute for Digital Engineering, University of Potsdam, Germany; 3 Experimental and Clinical Research Center, a joint cooperation between the Charité Universitätsmedizin Berlin and the Max Delbrück Center for Molecular Medicine in the Helmholtz Association, Berlin, Germany; 4 SRH Fernhochschule - The Mobile University, Riedlingen, Germany; 5 MRI.TOOLS GmbH, Berlin, Germany; 6 Medicinal Chemistry, Leibniz-Institut fϋr Molekulare Pharmakologie (FMP), Berlin, Germany; 7 Charité - Universitätsmedizin Berlin, corporate member of Freie Universität Berlin, Humboldt-Universität zu Berlin, and Berlin Institute of Health (BIH), Berlin, Germany

**Keywords:** Siponimod, Multiple Sclerosis, MRI, Fluorine, Molecular Imaging

## Abstract

Theranostic imaging methods could greatly enhance our understanding of the distribution of CNS-acting drugs in individual patients. Fluorine-19 magnetic resonance imaging (^19^F MRI) offers the opportunity to localize and quantify fluorinated drugs non-invasively, without modifications and without the application of ionizing or other harmful radiation. Here we investigated siponimod, a sphingosine 1-phosphate (S_1_P) receptor antagonist indicated for secondary progressive multiple sclerosis (SPMS), to determine the feasibility of *in vivo* ^19^F MR imaging of a disease modifying drug.

**Methods:** The ^19^F MR properties of siponimod were characterized using spectroscopic techniques. Four MRI methods were investigated to determine which was the most sensitive for ^19^F MR imaging of siponimod under biological conditions. We subsequently administered siponimod orally to 6 mice and acquired ^19^F MR spectra and images *in vivo* directly after administration, and in *ex vivo* tissues.

**Results:** The ^19^F transverse relaxation time of siponimod was 381 ms when dissolved in dimethyl sulfoxide, and substantially reduced to 5 ms when combined with serum, and to 20 ms in *ex vivo* liver tissue. Ultrashort echo time (UTE) imaging was determined to be the most sensitive MRI technique for imaging siponimod in a biological context and was used to map the drug *in vivo* in the stomach and liver. *Ex vivo* images in the liver and brain showed an inhomogeneous distribution of siponimod in both organs. In the brain, siponimod accumulated predominantly in the cerebrum but not the cerebellum. No secondary ^19^F signals were detected from metabolites. From a translational perspective, we found that acquisitions done on a 3.0 T clinical MR scanner were 2.75 times more sensitive than acquisitions performed on a preclinical 9.4 T MR setup when taking changes in brain size across species into consideration and using equivalent relative spatial resolution.

**Conclusion:** Siponimod can be imaged non-invasively using ^19^F UTE MRI in the form administered to MS patients, without modification. This study lays the groundwork for more extensive preclinical and clinical investigations. With the necessary technical development, ^19^F MRI has the potential to become a powerful theranostic tool for studying the time-course and distribution of CNS-acting drugs within the brain, especially during pathology.

## Introduction

Siponimod is one of the few drugs that show efficacy in secondary progressive multiple sclerosis (SPMS) patients 1. It is a next-generation sphingosine 1-phosphate receptor 1 (S_1_P_1_) and receptor 5 (S_1_P_5_) modulator. Siponimod is an oral treatment and is administered at a daily maintenance dose of 2 mg in SPMS patients 1. S_1_P_1_ receptor modulation reduces the egress of inflammatory lymphocytes from lymph nodes into the circulation, and infiltration into the central nervous system (CNS), where they cause MS pathology 2, 3. Several reports suggest that the therapeutic role of siponimod goes beyond inhibiting CNS inflammation via peripheral immunomodulatory mechanisms 4-6. Siponimod has potential S_1_P_1_ and S_1_P_5_ targets within the CNS, which might provide a mechanism for additional therapeutic action on brain pathology directly 7, 8.

CNS-acting drugs must cross the blood brain barrier (BBB) to exert their therapeutic effect 9. The BBB provides an important protective mechanism and controls the crossing of cells and toxins from the circulation into the CNS 10, but also presents a formidable challenge for delivery of therapeutic agents 11. The introduction of fluorine atoms enhances lipophilicity 12, promoting passive BBB permeation 13 and thus treatment efficacy 14, 15. Most CNS-acting small molecules such as neuroleptics and antidepressants are fluorinated, commonly with a trifluoromethyl (CF_3_) group, to facilitate BBB crossing 15, 16. Bioisosteric replacement with fluorinated moieties continues to be seminal in medicinal chemistry for drug discovery 15, 17 as well as tumor imaging 18.

Quantifying drug levels non-invasively would be instrumental for studying drug distribution especially in inaccessible organs such as the brain. Fluorine-19 (^19^F) magnetic resonance imaging holds the promise for mapping fluorinated drugs in the CNS. Since the amount of endogenous MR-detectable ^19^F atoms in the body is negligible, ^19^F MR imaging (MRI) and spectroscopy (MRS) offer a highly specific detection of administered fluorinated substances. Recently we employed ^19^F MRS to detect the fluorinated drug teriflunomide 19, an anti-inflammatory drug used for treating relapsing-remitting multiple sclerosis (RRMS).

Therapeutic actions and adverse reactions 20 can be attributed to the dose of drug accumulating in vulnerable organs. Drug levels are typically measured in blood or urine, even in the cerebrospinal fluid when clinically warranted, but this does not accurately reflect the potentially unevenly distributed drug levels within CNS tissue. Pharmacokinetic imaging is commonly conducted using autoradiography and single-photon emission computed tomography (SPECT) 21, which involve radioactive labelling methods that are unsuitable for routine clinical practice. ^19^F MRI is an additional tool for pharmacokinetic imaging that does not involve harmful ionizing radiation, making this approach particularly appealing 22, 23. A non-invasive method for localizing and quantifying fluorinated drugs such as siponimod in relevant organs during disease could allow for the integration of imaging and therapy, to establish a theranostic strategy that can tailor treatment to individual patient responses and pharmacokinetics 24, 25.

Many ^19^F MR applications have been conceptualized since the 1970s 26, 27. MRI of fluorinated drugs has been revisited multiple times, but low *in vivo* tissue concentrations have limited the number of successful applications 28. ^19^F MR imaging in the CNS has been previously achieved for drugs that are administered in very high dose regimes such as fluorinated anesthetics 29, 30 and for cytotoxic chemotherapies 31, 32. Disease-modifying drugs (DMDs) such as siponimod are typically present in much lower quantities and have not been imaged by ^19^F MRI thus far. Especially within the CNS, where only low drug levels are expected, low signal-to-noise ratios (SNR) pose a great challenge. Until now, only localized 33 or non-localized 19 ^19^F MR spectroscopy data - but not imaging - have been reported for other fluorinated drugs. ^19^F MRI will be an invaluable tool to clarify CNS acting mechanisms if these sensitivity limitations can be overcome.

In this study we characterized the MR properties of siponimod and investigated MR methods tailored for the acquisition of short T_2_/T_2_^*^ siponimod. We identified ultrashort echo time (UTE) MRI as the most sensitive method to acquire siponimod images under biological conditions and show the feasibility of localizing siponimod with *in vivo* ^19^F MRI in a mouse model. For the first time, we could image and localize the signal of siponimod *in vivo* in the stomach and liver, after oral administration. *Ex vivo* investigations revealed siponimod in the kidney, brain, and thymus, even after a single dose. We acquired 3D images of siponimod in liver and brain *ex vivo* and observed varying levels of the drug within the tissue. To investigate the potential for preclinical *in vivo* imaging in the brain and for future clinical translation, we also acquired images with shortened protocols at lower spatial resolution, and compare the sensitivity achieved with state-of-the-art radio-frequency (RF) hardware for imaging the mouse brain at 9.4 T versus imaging the human brain at 3.0 T, a commonly used clinical field strength. Finally, we estimated the concentration of siponimod reached in a mouse brain.

## Methods

### Siponimod and phantoms

Siponimod (BAF312) was purchased from MedChemExpress LLC (New Jersey, USA) and dissolved in 100% dimethylsulfoxide (DMSO, Roth; Karlsruhe, Germanyl) at a concentration of 58 mM (29.4 mg/ml) or in 100% human serum (from male type AB plasma, Sigma-Aldrich, H4522) at a concentration of 6.8 mM or 3.9 mM (3.5 mg/ml or 2 mg/ml) for *in vitro* experiments, and formulated in 0.6% carboxymethylcellulose (CMC, Sigma, Schnelldorf, Germany) as stabilizer and suspending agent at a concentration of 19.4 mM (10 mg/ml) for *in vivo* administration. The latter was formulated using a vortex mixer and kept at body temperature prior to application. The MR properties of siponimod were characterized in 2 ml phantoms: syringes (inner diameter (ID) of 10 mm) equipped with stopper closing-cones (B. Braun, Melsungen, Germany) and filled with either the solution in DMSO or in serum (6.8 mM). For continuous, homogenous heating at physiological temperature (PT, 37 °C), phantoms were inserted into a coil of tubing with circulating water; temperature was monitored with a fiber-optic sensor and controlled with a remote water bath. The same phantom was employed for the comparison of RARE, FLASH, bSSFP and UTE pulse sequences. A 10 ml syringe phantom (ID = 16 mm) with 3.9 mM siponimod dissolved in serum was used to determine the relative sensitivity of the *ex vivo* liver and brain imaging protocols and as a reference tube for determining the siponimod concentration.

To compare between imaging a human brain at 3.0 T and a mouse brain at 9.4 T, two phantoms mimicking the respective loading characteristics were built. An outer compartment was filled with a mixture of distilled water, sucrose and NaCl matching the electromagnetic properties of gray matter (40%) and white matter (60%) at 123 MHz or 400 MHz (123 MHz: 1041 g sucrose and 25 g NaCl, 400 MHz: 1420 g sucrose and 43 g NaCl per 1l water) 34, 35. A smaller inner compartment was filled with distilled water only, to provide a volume with identical proton density. For both phantoms, 0.073% CuSO_4_ was added to both compartments to shorten T_1_ relaxation and prevent bacterial growth. For imaging at 3.0 T, we used a 2800 ml sphere with a 15 ml polypropylene conical tube (ID = 14 mm, Corning Science México, Raynosa, México) insert. At 9.4 T, we used a syringe (ID = 16 mm, B. Braun, Melsungen, Germany) filled to 12 ml with a glass NMR tube (ID = 3 mm, Wilmad-Labglass, Vineland, USA) as an insert.

### Animal experiments

All animal experiments were conducted in accordance with procedures approved by the Animal Welfare Department of the State Office of Health and Social Affairs Berlin (LAGeSo) and conformed to guidelines to minimize discomfort to animals (86/609/EEC).

### *In vivo* experiments

Heathy male (n = 5, designated M1, M2, M4-M6) and female (n = 1, M3) C57BL/6 mice (4-6 months old) were anesthetized by intraperitoneal (IP) injection using a mixture of xylazine (5 mg/kg, CP Pharma, Burgdorf, Germany) and ketamine (50 mg/kg, WDT, Garbsen, Germany) maintained by an IP catheter line. After achieving the appropriate level of anesthesia, an intubation catheter was inserted into the esophagus of the mouse for later administration of siponimod suspension (the distance to the stomach was measured and marked on the catheter prior to insertion). Mice were transferred to a temperature-regulated animal holder and supplied with pressurized air (30%) and O_2_ (70%). A respiration probe and body temperature sensors (Neoptix, OmniLink version 1.15, Omniflex, Neoptix, Québec, Canada) were connected for continuous monitoring of physiological parameters and the body temperature was maintained throughout the measurements. The animal holder was inserted into the MR scanner bore and Cryogenic Radiofrequency Probe (CRP, see below). A single dose of siponimod (4 mg in 400 μl CMC, ≈133 mg/kg body weight) was administered remotely to each mouse while in the scanner during the MR measurements (see below).

### Preparation of *ex vivo* samples

Mice were transcardially perfused with 20 ml phosphate-buffered saline (PBS) followed by 20 ml paraformaldehyde (4% PFA, Santa Cruz Biotechnology, Inc., Dallas, TX, USA) at the end of each *in vivo* experiment 36. *Ex vivo* tissues including liver, kidneys, brain, and thymus were extracted and stored in tubes filled with 4% PFA. After fixation, the tissues were embedded in 2% agarose in 15 ml conical centrifuge tubes (liver, kidneys) or 5 ml Eppendorf tubes (brain, thymus) to prevent movement during the MR acquisition. All samples were stored at 4 °C.

### MR hardware

With the exception of sensitivity comparison measurements at 3.0 T, all MR experiments were performed on a Biospec 9.4 T horizontal bore MR scanner (Bruker Biospin, Ettlingen, Germany). We used both an in-house built room temperature (RT, 20 °C) dual-tunable ^19^F/^1^H head RF volume coil (ID = 16 mm) 37 and a cryogenically-cooled 2-channel transceive ^19^F quadrature RF surface probe (ID = 20 mm, Cryogenic Radiofrequency Probe or CRP, Bruker, Fällanden, Switzerland) 36. Since the ^19^F CRP does not have a ^1^H channel, anatomical ^1^H scans were performed with a linear ^1^H volume coil (ID = 72 mm, Bruker Biospin, Ettlingen, Germany) using a CRP replica emulating the geometry of the CRP coil-head and supporting components to reproduce the position in the CRP.

Due to the very close gyromagnetic ratios of the ^1^H and ^19^F nuclei (≈6% deviation) 38, conclusions about RF hardware aspects of MR sensitivity obtained at the ^1^H resonance frequency are transferable to ^19^F measurements. For the comparison of preclinical and clinical settings, we used a ^1^H cryogenic quadrature RF probe (Bruker, Fällanden, Switzerland) with a very similar geometry as a proxy for the ^19^F CRP 36, 39. The measurements at 3.0 T were performed on a Siemens Magnetom SkyraFit (Siemens Healthcare, Erlangen, Germany) using the built-in body RF array for transmission and a 32-channel head RF array (Siemens Healthcare, Erlangen, Germany) for reception.

## MR protocols

### MR characterization experiments

The ^19^F MR properties of siponimod were characterized in DMSO and in human serum at RT and PT. They were also measured in *ex vivo* liver tissue at RT. All protocols were performed with the RT head volume RF coil and used Gauss excitation RF pulses with a bandwidth (BW_RF_) of 10 kHz and a spectral acquisition bandwidth (BW_spec_) of 200 kHz. We used non-localized free induction decay (FID) MRS sequences with 13 different repetition times (TR) to determine T_1_. The DMSO RT and DMSO PT protocols used: TR = 136 - 5000 ms, acquisition time (TA) =4 min for each TR, acquired points (n_acq_) = 4096. Serum RT and serum PT protocols used: TR =20 and 2500 ms, TA = 20 min, n_acq_ = 1024 (RT) or 2048 (PT). For the ^19^F MRI assessment of the *ex vivo* samples we used: TRs=20-2500 ms, TA = 3 h, and n_acq_ = 1024. T_2_ was measured with Carr-Purcell-Meiboom-Gill (CPMG) MR spectroscopy (number of echoes = 32). The measurements in DMSO used TR = 5000 ms, ΔTE = 32 ms (RT) or 41.5 ms (PT), TA = 10 min, and n_acq_ = 6144 (RT) or 8192 (PT). In serum, we used TR = 2500 ms, ΔTE = 3.2 ms, TA = 20 min (RT) or 1 h (PT), and n_acq_ = 512. For the *ex vivo* samples, we conducted a 70 h measurement with TR = 1000 ms, ΔTE = 3.2 ms, and n_acq_ = 512.

To determine the most sensitive imaging method, we acquired 3D images of siponimod in the serum phantom with RARE, FLASH, bSSFP and UTE MRI techniques, which were optimized based on the determined T_1_ and T_2_ relaxation times. Measurements were performed at RT and PT with the head volume RF coil. The RT experiments were repeated with the CRP. For all protocols, the field of view (FOV) was 28×28×28 mm^3^, image matrix 32×32×32 voxels, TA = 4 h (20 min with CRP), and receiver bandwidth (BW_read_) =75 kHz. RARE was employed with flip-back pulse and centric phase encoding. ΔTE was set to the shortest possible timing (1.5 ms). Based on the measured transverse relaxation times, we calculated the expected point spread function (PSF) in the phase encoding direction and chose the highest ETL with a full width at half maximum (FWHM) of the PSF below 1.5 voxels (ETL = 8 at RT, ETL = 16 at PT) 40, 41. TR was optimized based on the steady-state signal equation (TR = 350 ms at RT, TR = 369 ms at PT) 42. The FLASH sequence was used with the minimal possible TE (0.89 ms), a short TR (10 ms), and the Ernst excitation flip angle (α = 15.2° at RT, α = 14.2° at PT) 42. For bSSFP, TR = 1.4 ms was chosen and the excitation angle set to arccos((T_1_/T_2_ - 1)/(T_1_/T_2_ + 1)) (α = 16.5° at RT, α = 26.3° at PT) 43. UTE was optimized as the FLASH sequence (TR = 10 ms, TE of 0.14 ms, α = 15.2° at RT and α = 14.2° at PT). In each case, a separate noise scan was acquired with 1 average and 0 W transmit power.

### *In vivo* experiments

All *in vivo* ^19^F measurements were performed with the CRP positioning the mouse abdomen at the center of the coil. One non-localized ^19^F MR spectrum (TR = 1000 ms, block pulse, BW_RF-excitation_ = 10 kHz, BW_receiver_ = 25 kHz, n_acq_ = 4096, TA = 128 s) was acquired before the administration of siponimod. Directly following the administration, an interleaved series of ^19^F MRS acquisitions (as above) and 2D-UTE experiments (3 horizontal slices, 6 mm slice thickness, 32×32 mm^2^ FOV, 32×32 voxel image matrix, TR = 100 ms, block pulse, BW_RF-excitation_ = 10 kHz, TE = 0.27 ms, α = 28°, BW_receiver_ = 20 kHz, TA = 10 min) was started. Depending on the stability of the anesthesia, the protocol was repeated 10, 19 or 9 times for mouse M1, M2 or M3, respectively. During analysis, 3 acquisitions were averaged, corresponding to a ^19^F TA of 30 min. Multiple ^1^H images were measured in between to control for shifts in the animal's position (not shown). After the ^19^F acquisitions, the animal was transferred to the 72 mm ^1^H volume RF resonator to acquire a high-quality anatomical image (RARE, 20 horizontal slices, slice thickness = 0.9 mm, FOV = 30×30 mm^2^, matrix size 154×154, TR = 1200 ms, TE = 5.9 ms, BW_receiver_ = 81.5 kHz, TA = 10 min).

### *Ex vivo* experiments

We acquired ^19^F spectra of all *ex vivo* tissue samples using the CRP to detect siponimod and possible metabolites: non-localized MRS, TR = 50 ms, block pulse, BW_RF-excitation_ = 200 kHz, BW_receiver_ = 200 kHz, n_acq_ = 512, α = 30°, TA = 1 h. This protocol was repeated 18 times spaced over 3 days without any sample inserted to characterize short T_2_^* 19^F signals originating from the RF coil itself.

3D-UTE images were acquired for liver (M1-M3) and brain (M4-M6) tissue samples. In both cases, the CRP was used to achieve sufficient sensitivity and the center RF resonance frequency was offset from the siponimod resonance frequency by 2 kHz to avoid exciting nuisance signal from the RF coil. Acquisition parameters for the liver were: FOV = 28×28×28 mm^3^, matrix size = 32×32×32, TR = 10 ms, Gauss pulse, BW_RF-excitation_ = 10 kHz, TE = 0.14 ms, α = 14°, BW_receiver_ = 75 kHz, TA = 16 h (3632 averages), 2-fold radial undersampling. For the brain we used the same parameters, except FOV = 24×24×24 mm^3^, matrix size = 24×24×24 and TA = 64 h (27024 averages). In both cases, additional scans with 1 average and 0 W transmit power, but with otherwise identical settings were acquired to estimate the noise level, necessary for SNR estimation and quantification. To determine the relative sensitivity of these ^19^F 3D-UTE protocols, identical measurements were performed on the 10 ml serum phantom with 1362 averages (liver protocol) and 844 averages (brain protocol). Anatomical images were recorded similar to the *in vivo* experiment for both the liver (RARE, 18 horizontal slices, slice thickness = 0.87 mm, FOV = 28×28 mm^2^, matrix size = 512×512 , TR = 2500 ms, TE = 13.5 ms, BW_receiver_ = 75 kHz, TA = 32 min) and the brain (RARE, 12 horizontal slices, slice thickness = 1 mm, FOV = 24×24 mm^2^, matrix size = 168×168 , TR = 2500 ms, TE = 22 ms, BW_receiver_ = 35 kHz, TA = 12 min).

To demonstrate the possibility of localizing siponimod with a shorter acquisition time, additional *ex vivo* images were acquired of the brain of M6. The above 3D-UTE brain protocol was adapted by removing radial undersampling, increasing the FOV to 48×48×48 mm^3^ or 64.8×64.8×64.8 mm^3^ for 2 mm or 2.7 mm isotropic resolution, and reducing the TA to 60 min (205 averages) or 10 min (35 averages), respectively. The acquisition at 2 mm resolution was repeated 4 times and the average used for estimating the concentration of siponimod. A corresponding image of the 10 ml syringe phantom (3.9 mM siponimod in serum) was acquired with 30 averages as a SI reference. The anatomical image^ 1^H protocol was adapted by matching the slice thickness to the new ^19^F resolutions.

### Sensitivity comparison to clinical imaging at 3.0 T

The human brain is approximately 2750 times larger than the mouse brain (V = 1400 cm^3^ vs. V = 0.509 cm^3^) 44, 45. Thus, equivalent relative spatial resolution is achieved with 2750 times larger voxel volumes, which is equal to 14 times longer voxel edge lengths for 3D isotropic imaging. We used Cartesian 3D gradient echo protocols with adapted FOVs but otherwise identical parameters: matrix size = 256×256×16, TR = 3000 ms (full relaxation), TE = 2.8 ms, α = 90°, BW_receiver_ = 200 kHz, 1 average. The FOVs were 358×358×22.4 mm^3^ and 25.6×25.6×1.6 mm^3^ for 3.0 T and 9.4 T, respectively.

### Data analysis and image reconstruction

All data analysis was performed using MATLAB and Optimization Toolbox Release 2018a (The MathWorks, Inc., Natick, Massachusetts, United States).

### MR spectroscopy processing

All chemical shifts reported are referenced to trichloro-fluoro-methane (CFCl_3_).

The FID-sequence data of the MR characterization, acquired with the 1-channel volume coil, was processed by removing the digital filter delay and zero-padding the time domain data to 16384 points before Fourier transformation. 0^th^ order phase correction was performed manually and a real Voigt peak with baseline was fitted to the spectrum using Matlab's lsqnonlin routine and the trust-region-reflective algorithm to quantify the signal intensity (SI) 46-48. To quantify the ^19^F T_2_^*^ of siponimod in DMSO or serum and *ex vivo*, the spectrum was transformed back to the time domain after phase correction and shifting the signal peak to the central frequency. Afterwards, the phase corrected FID signal was cut to the original length. The echoes of the CPMG data were individually Fourier transformed without zero-padding and the 0^th^ order phase was corrected manually before computing the SI by integrating the real signal over a width of 1.5 kHz (DMSO) or 5 kHz (serum and *ex vivo*) around the peak.

The *in vivo* and *ex vivo* MRS data acquired with the 2-channel CRP was prepared in a similar fashion. The time domain data of the *in vivo* experiments and the control experiments with an empty RF coil was truncated to 256 points and that of all *ex vivo* measurements to 768 points before Fourier transformation to increase SNR. Zero-padding was limited to 4096 points to accelerate fitting procedures. Further processing steps of the control experiment and *ex vivo* data are described in the following section. For the *in vivo* data these steps were not necessary due to the shorter measurement time and thus lower sensitivity. Here complex Voigt peaks were first fitted to each channel individually to estimate 0^th^ and 1^st^ order phase correction terms. After application of the phase correction, the channels were averaged and a Voigt peak was fitted to the combined spectrum to estimate the SI.

### Artifact signal characterization and subtraction

**Figure 1:**
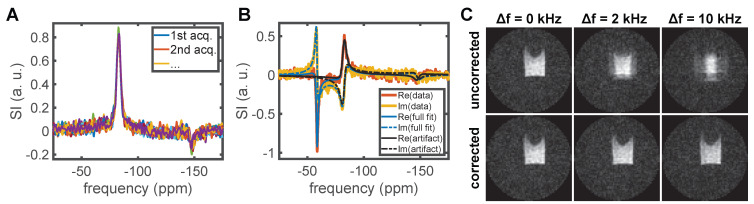
^19^F traces in the RF coil were detected with spectroscopy protocols optimized for short T_2_^*^ compounds. Here the real part of 18 spectra acquired with an unloaded RF coil is shown with manually adjusted 0^th^ and 1^st^ order phase (A). The two ^19^F lines occur at ≈-83 ppm and ≈-147 ppm. A. u. denotes arbitrary units. To remove this artifact from spectra acquired in *ex vivo* tissue samples, the sum of three complex Voigt functions was fitted with Bayesian constraints on the two artifact lines (B). The artifact component could then be subtracted. We show the full fit and artifact component for the channel 1 data of kidney M2. See Figure 5 for the final result. To selectively excite only the siponimod resonance in UTE imaging while minimizing the excitation pulse length, off-resonance excitation shifted by +2 kHz was used. The resultant image blurring was corrected by multiplication with a delay-dependent phase factor. (C) shows the efficacy of this correction for a 3D UTE protocol with 64×64×64 image matrix and data acquired in a phantom with siponimod dissolved in DMSO.

^19^F MR spectra acquired with the empty CRP containing no sample showed two distinct lines at ≈-83 ppm and ≈-147 ppm (Figure 1A). Such artifact signals can originate from trace amounts of fluorinated substances in, for example, capacitor electrolytes and lubricants used for the manufacturing of the RF coil. Contamination of MR spectra could be mitigated by selective excitation, suppression techniques, or in post-processing. We opted for the latter by fitting an analytic line shape model and subtracting the artifact contribution to preserve any potential metabolite signals. The code used for this is available on Github and details can be found in the associated documentation 49.

The artifact was characterized by fitting two complex Voigt peaks to the 18 spectra acquired with an empty RF coil 48, 50, 51. Each RF channel of the data was treated separately. The model was parameterized with coupled peak amplitudes to later enable a Bayesian constraint on the relative contributions independent of the absolute SI: L(f) = a⋅e^-iϕf^⋅(r⋅e^-ip^⋅V(f,v_1_,w_1_,m_1)_ + (1-r)⋅e^-iq^⋅V(f,v_2_,w_2_,m_2)_), where L is the modeled line shape, f the frequency, a the joint amplitude, ϕ the first order phase correction, r the peak ratio, p and q are the zeroth order phase correction, and V is a complex Voigt function with center frequency v, full widths at half maximum (FWHM) w and mixing parameter m. To enable automatic fitting, Weideman's polynomial approximation of the error function was used for fast computation of the Voigt function 49, 52. Based on the 18 acquired control spectra (Figure 1A), we found the following means and standard deviations: for channel 1 r = 0.84±0.02, v_1_ = -82.96±0.08 ppm, w_1_ = 5.1±0.4 ppm, r_1_ = 0.82±0.06, v_2_ = -147.9±0.4 ppm, w_2_ = 6±1 ppm, r_2_ = 0.8±0.2; for channel 2 r = 0.85±0.01, v_1_ = -82.5±0.1 ppm, w_1_ = 4.8±0.3 ppm, r_1_ = 0.79±0.06, v_2_ = -146.9±0.4 ppm, w_2_ = 5±1 ppm, r_2_ = 0.7±0.2.

The knowledge of this artifact signal was then used to constrain fits of the acquired spectra of *ex vivo* tissue samples by placing Gaussian priors on two of three modeled peaks. To account for magnetic field drift and inhomogeneity, the uncertainty on the artifact frequencies was increased equivalent to the addition of a zero mean Gaussian variable with a standard deviation of 250 Hz. The third peak was left unconstrained and initialized to capture the main siponimod signal. To remove the artifact signal, the corresponding portion of the fit was subtracted (Figure 1B). Due to the limited degrees of freedom of the model, it is expected that potential metabolite signals are not captured by the fit and thus unaffected. Analogous to the processing of the *in vivo* data, a single, unconstrained complex Voigt peak was fitted to the cleaned spectrum to estimate the 0^th^ order phase term. After phase correction, the data of both RF channels was averaged, and the noise level was determined in a background region of the combined spectrum. Both the peak signal-to-noise ratio (pSNR) and the ratio of the area under the curve of a fitted Voigt peak to the noise level (area-to-noise ratio, ANR) are reported as metrics of SI.

### MR Relaxometry

We computed T_1_, T_2_ and T_2_* relaxation times using a non-linear least squares cost function, the Levenberg-Marquardt algorithm and Matlab's lsqcurvefit function. The parameter covariance matrix was estimated using the linear approximation: V_p_ = σ_r_^2^(J^T^J)^-1^, where J denotes the Jacobian and σ_r_^2^ the error variance calculated from the residuals 53, 54. We report parameter standard errors given by the square root of the diagonal elements of V_p_. The used SI equations were S(TR) ∝ 1 - exp(-TR/T_1_) for T_1_ estimation and both S(t) ∝ exp(-t/T_2_) and S(t) ∝ β⋅exp(-t/T_2_^(a)^) + (1 - β)⋅exp(-t/T_2_^(b)^) for T_2_ and T_2_^*^ estimation. As the mono-exponential decay can be understood as a constrained version of the bi-exponential model, a likelihood ratio test was performed to determine whether the hypothesis that T_2_ or T_2_^*^ decay follow a single exponential curve can be rejected. Apparent T_2_ and T_2_^*^ values summarizing the bi-exponential decay were calculated by sampling 5⋅10^4^ parameter values from a multivariate normal with mean equal to the parameter estimate and covariance V_p_, determining t^'^ where S(t^'^) = S(0)⋅exp(-1) for each sample, and reporting the mean and standard deviation of the results for t'.

### Image reconstruction and analysis

UTE images were reconstructed using the Michigan Image Reconstruction Toolbox (MIRT) non-uniform fast Fourier transform (NUFFT) based on trajectory measurements performed with a ^1^H coil and an adjusted FOV to compensate the change of gyromagnetic ratio 55-58. For data acquired with the CRP, each channel was reconstructed separately before computing a root sum-of-squares image. Off-resonance effects in the *ex vivo* imaging data were compensated before image reconstruction by multiplying each k-space point with delay-dependent phase factor exp(2πi⋅(TE + (j-1)⋅t_d_)⋅Δf), where i is the imaginary unit, TE the echo time, j the position on the k-space spoke, t_d_ the frequency encoding dwell time and Δf the off-resonance frequency shift (Figure 1C). Noise levels were estimated based on a background region (*in vivo*) or a separate noise scan (*ex vivo*) and background subtraction was performed with cluster-based thresholding 59 at SNR = 3.5 (*in vivo*) or 4.0 (*ex vivo*) following the protocol outlined in Starke et al. 60.

For comparison of the employed *ex vivo* liver and brain protocols, SNR maps of the serum phantom measurements were adjusted for the difference in scan time to the *ex vivo* acquisitions, the FOVs and spatial resolutions of the SNR maps were matched by nearest neighbor interpolation and the SNR ratio was averaged over all voxels with SNR > 10 in both images. To estimate the siponimod concentration in a mouse brain, the pixelwise SI ratio between data acquired in the brain and in the reference phantom was computed, and the result multiplied by the reference concentration.

### Sensitivity comparison to clinical imaging at 3.0 T

Noise data was extracted from rectangular 32×32×16 voxel background regions located in the 4 corners of the individual image volumes obtained for each RF receive channel. Noise prewhitening was performed with Cholesky decomposition of the inverse noise covariance matrix followed by sum-of-squares reconstruction 61, 62. The SI was evaluated in a central slice of the phantom by averaging over 32×5 (3.0 T) or 12×12 (9.4 T) voxel rectangles inside the inner compartment. SNRs were determined taking into account the RF channel number-dependent noise distributions 60, 63.

## Results

### Temperature and protein binding alter the MR properties of siponimod

**Figure 2:**
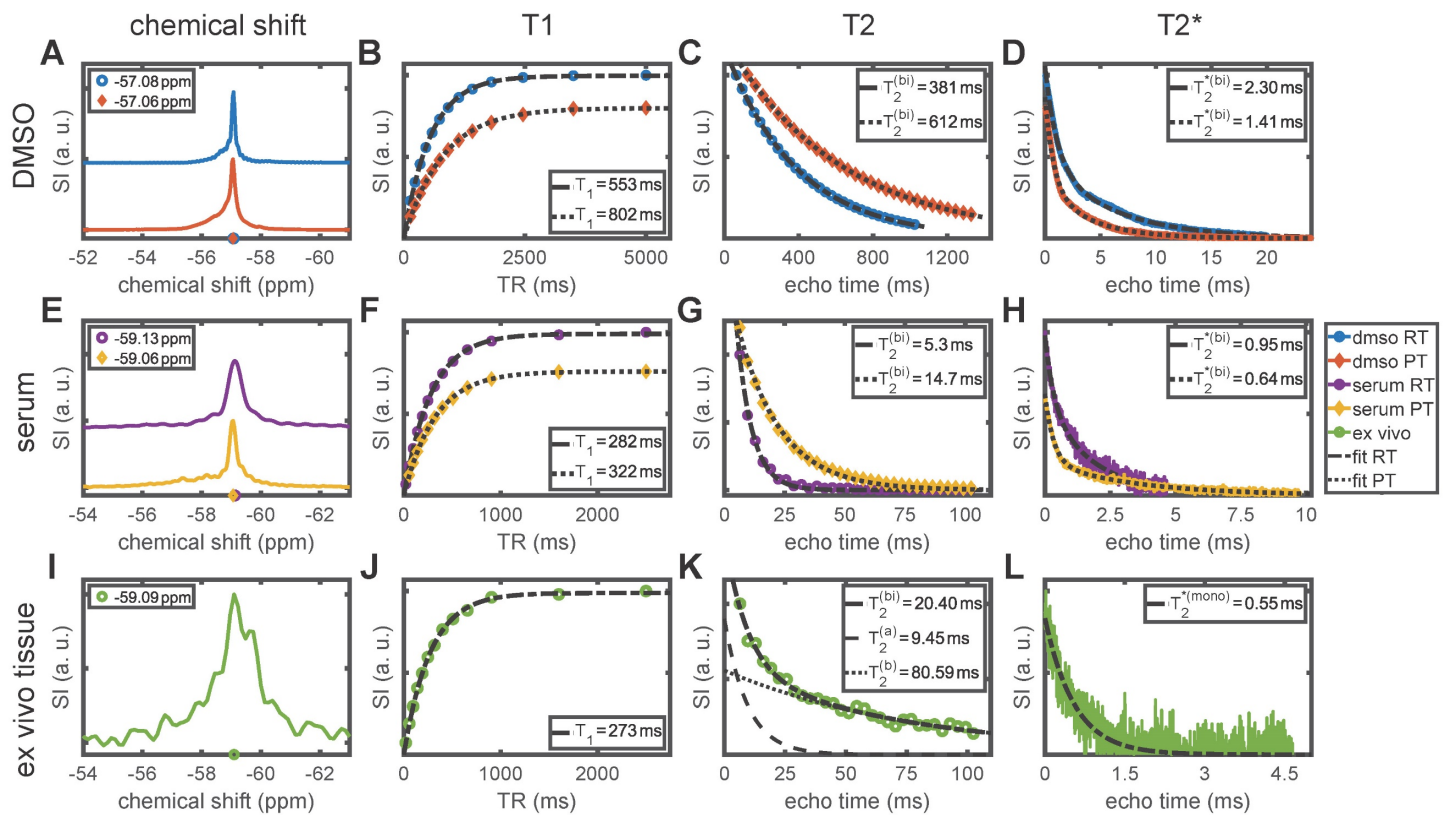
** Chemical shift and MR relaxation times for siponimod dissolved in DMSO and serum (mimicking biological conditions), and in *ex vivo* liver tissue from mouse M3.** Room temperature (RT) equals 20 °C and physiological temperature (PT) 37 °C. Where the hypothesis of a mono-exponential decay was rejected, an apparent T_2_ or T_2_^*^ value (T_2_^(bi)^ or T_2_^*(bi)^) is reported, which reflects the time after which 63% of the signal has decayed. More detailed results, including measurement uncertainties, are reported in Table 2.

*In vitro* and *ex vivo* experiments showed that the ^19^F MR properties of siponimod are strongly influenced by the chemical environment. All results are outlined in Table 2 and Figure 2. Siponimod gives a single peak spectrum under the investigated conditions. In DMSO, a chemical shift of -57.08 ppm (at RT ≈ 20 °C) and -57.06 ppm (at PT ≈ 37 °C) was observed (Figure 2A). In the presence of plasma protein (in serum), the resonance frequency shifted to -59.13 ppm (RT) and -59.06 ppm (PT, Figure 2E). A similar observation was made in *ex vivo* liver tissue, where a chemical shift of -59.09 ppm was observed at RT.

Longitudinal relaxation time measurements in DMSO yielded T_1_ = 553±4 ms (RT) and T_1_ = 802±7 ms (PT, Figure 2B). In serum, T_1_ was shortened to 282±2 ms (RT) and 322±2 ms (PT, Figure 2F), which is similar to 273±4 ms observed in *ex vivo* tissue at RT (Figure 2J). For transverse relaxation, the shortening of relaxation times in serum and *ex vivo* tissue compared to DMSO was even more pronounced (up to 98% reduction). For all conditions, the null-hypothesis (mono-exponential decay) was rejected in favor of a bi-exponential decay (P ≪ .001, Table 2). We observed an apparent T_2_ of 381 ms (RT) and 612 ms (PT, Figure 2C) in DMSO. In serum, T_2_ was 5.3±0.4 ms (RT) and 14.7±0.4 ms (PT, Figure 2G). *Ex vivo*, a bi-exponential decay with an apparent T_2_ of 20±3 ms was observed (Figure 2K, see Table 2 for details). For T_2_^*^, a bi-exponential decay also best described the FID signal in DMSO and in serum (P ≪ .001, Table 2). We found apparent T_2_^*^ = 2.30 ms (DMSO RT), 1.41 ms (DMSO PT), 0.95±0.01 ms (serum RT), and 0.643±0.007 ms (serum PT). For the *ex vivo* sample, the mono-exponential decay hypothesis was not rejected (P = .09) and we observed T_2_^*^ = 0.55±0.02 ms. The stated uncertainties reflect the ambiguity of the fits only, and no deviation is given where the value falls below 1%, as in those cases divergences due to biological factors or temperature variations can be expected to dominate.

### Ultrashort echo time MRI is most sensitive for imaging siponimod

**Figure 3:**
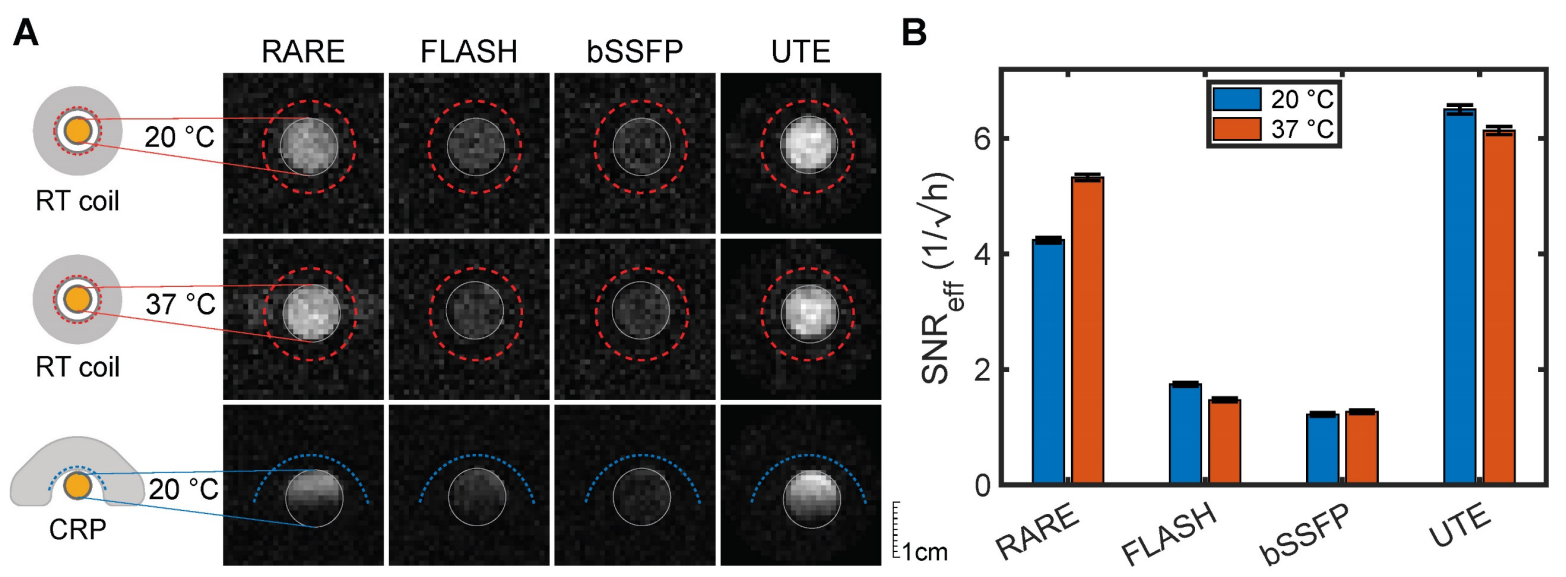
** Comparison of optimized 3D ^19^F MR protocols imaging the serum phantom.** (A) The first two rows show measurements with the room temperature (RT) volume RF coil at sample temperatures of 20 °C (RT) and 37 °C (PT). The acquisition time was 4 h per image. The third row highlights the different impact of the surface coil design of the CRP (20 min acquisition time). Each row is adjusted to one SNR scale. (B) SNR efficiencies (SNR/√(acquisition time)) in the volume RF coil measurements. Error bars show the standard error of the measurement (numerical values are reported in Table 3). UTE showed superior SNR_eff_ compared to the other three sequences for both temperature conditions (p_UTE-RARE, RT_ = 3.7⋅10^-19^, p_UTE-RARE, PT_ = 1.5⋅10^-133^, p-value below 2.3⋅10^-308^ for all other comparisons, two-sided z-test).

As the relaxation times in serum closely mimicked those observed *ex vivo* and were also expected to approximate *in vivo* conditions, optimized MRI acquisition methods were compared by experiments on a serum phantom. At both RT and PT, measurements using a volume transceive RF coil with high transmission and reception field (B_1_) homogeneity showed the highest SNR_eff_ for the UTE protocol (Figure 3A and B, Table 3). FLASH and bSSFP were much less SNR efficient with more than 70% lower SNR_eff_. RARE emerged as the next best MRI method with 36% and 13% lower SNR_eff_ than UTE at RT and PT, respectively (Table 3).

Analogous experiments using a ^19^F cryogenically-cooled surface transceiver RF coil (CRP, see Methods) showed similar differences between the MRI methods (Figure 3A, lower panel) but highlight inhomogeneity and signal loss distal from the coil, characteristic of surface transceiver soils. The RARE protocol achieved high sensitivity only in the region close to the RF coil array, while FLASH and UTE had a much better signal coverage. Based on the peak SNR measured with UTE and the CRP (Table 3), we can extrapolate that siponimod concentrations as low as 516 µM would be detectable under ideal conditions at the tested spatial resolution, with an acquisition time of 1 h. This is equivalent to 6.2⋅10^14 19^F-atoms per voxel. Importantly, the detection limit can be further reduced by increased averaging or with larger voxel sizes. It is inversely proportional to the square root of the measurement time. For radial sequences such as UTE, the SNR does not strictly follow the same straightforward dependence on the image resolution as with Cartesian sampling. Thus, we determined experimentally the detection limits for two lower spatial resolution protocols: at 2 mm and 2.7 mm 3D isotropic spatial resolution, the detection limit with 1 h of measurement time dropped to 76 µM and 33 µM, respectively. This equates to 186 µM and 82 µM detection limits for a 10 min acquisition.

### *In vivo* ^19^F MRS and MRI of siponimod in the stomach and liver

**Figure 4:**
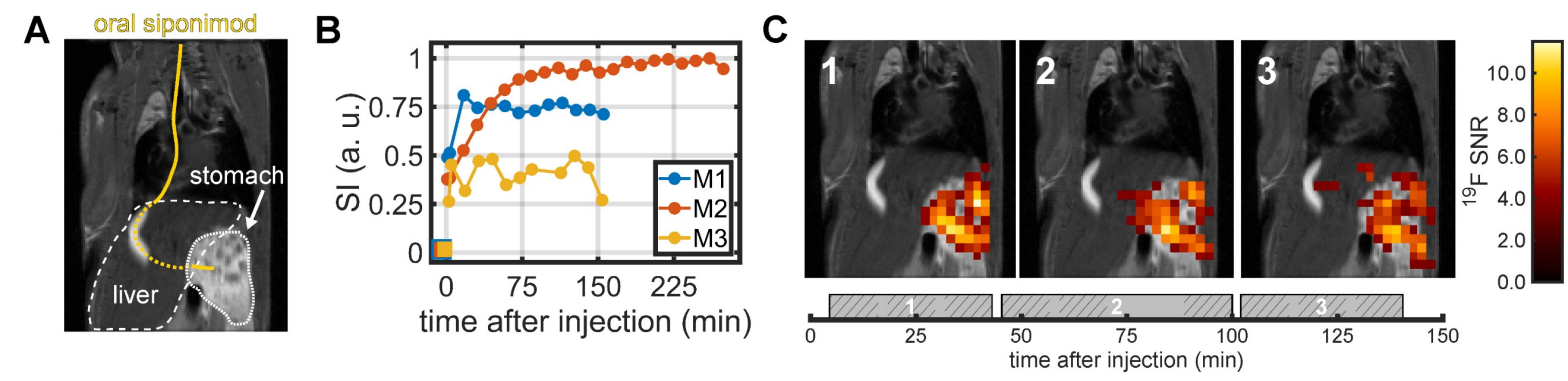
***In vivo* ^19^F MRS and MRI after oral administration of siponimod.** (A) Siponimod suspended in carboxymethylcellulose was administered through an oral intubation tube (marked in yellow) directly into the stomach. (B) Dynamic tracking of non-localized ^19^F MRS signal intensity. (C) 2D ^19^F UTE images overlaid on an anatomical ^1^H scan. Each image corresponds to the average of 3 × 10 min ^19^F acquisition. The time envelope of these is indicated by the gray boxes in the bottom plot with the hashed areas highlighting the actual UTE measurement.

We remotely administered siponimod via an intubation catheter to the stomach (Figure 4A) and acquired ^19^F MRS measurements interleaved with ^19^F imaging using the CRP. A ^19^F MRS signal was detected within the first 5 min following siponimod administration in all three mice, after which a signal increase was observed over the initial minutes of the experiment (Figure 4B). The increase was much faster for mice M1 and M3 than for mouse M2, for which the signal took more than an hour to gradually reach a plateau. In all mice, the MR spectroscopy signal was mostly stable throughout the measurement, indicating a prolonged gastric emptying and slow uptake into the blood stream. Mouse M3 showed a sharp signal drop after 120 min, which coincided with an increase in the respiration rate, indicating attenuation of anesthesia, that required termination of the experiment (Figure 4B).

A clearly localized siponimod-derived ^19^F signal was observed in the stomach. The signal mostly remained stable over time (Figure 4C). The second and third imaging block (45-100 min and 102-145 min after siponimod administration) showed ^19^F signal also in the liver. However, the highest SNR values were observed only within the stomach (Figure 4C).

### Siponimod-derived signal is detected in *ex vivo* tissues

**Figure 5:**
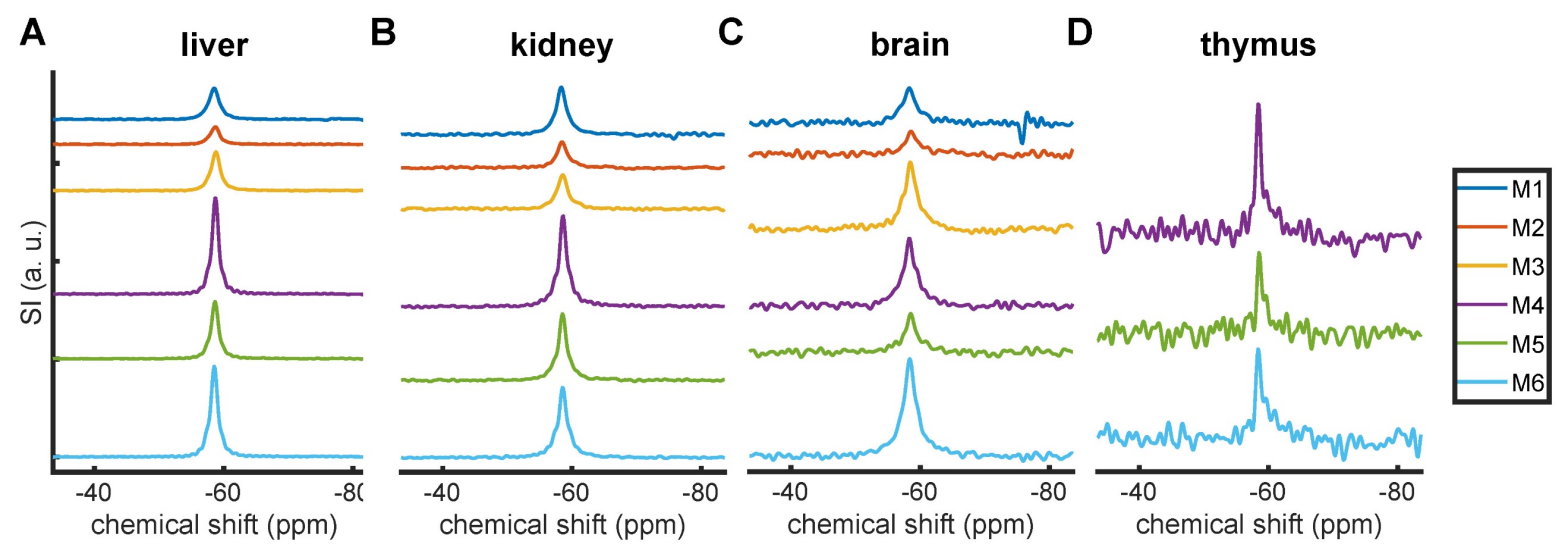
**^19^F MRS in *ex vivo* tissues. Signal intensities represent the SNR with one common scale for each organ.** Mice M1-M3 underwent perfusion ≈2.5-4 h after siponimod administration while M4-M6 were perfused after ≈6 h.

We acquired *ex vivo* ^19^F MR spectra from liver, kidney, brain, and thymus samples from 6 mice. A siponimod-derived ^19^F MR signal was detected in all samples (Figure 5). Due to the inhomogeneous transmission and reception field of the CRP surface coil, SNRs and ANRs can be considered only semi-quantitative measures of the amount of siponimod in the sample. The highest SNRs were observed in the liver, followed by the kidney (Figure 5A and B, Table 4). All samples exhibited a single peak with a chemical shift of -59 ppm similar to the spiked serum (Figure 2). The signal from the brain of mouse M1 showed a secondary peak (Figure 5C). Only weak signals were detected in the thymus (Figure 5D).

Mice M4-M6 showed increased ^19^F MR signals in all tissues, due to a longer time after oral administration (Table 4). Yet even in the same cohort, large variations were seen, for example in the observed SNRs for the liver and brain between M2 and M3 (>100% difference).

### *Ex vivo* ^19^F MRI of siponimod in the liver and brain

**Figure 6:**
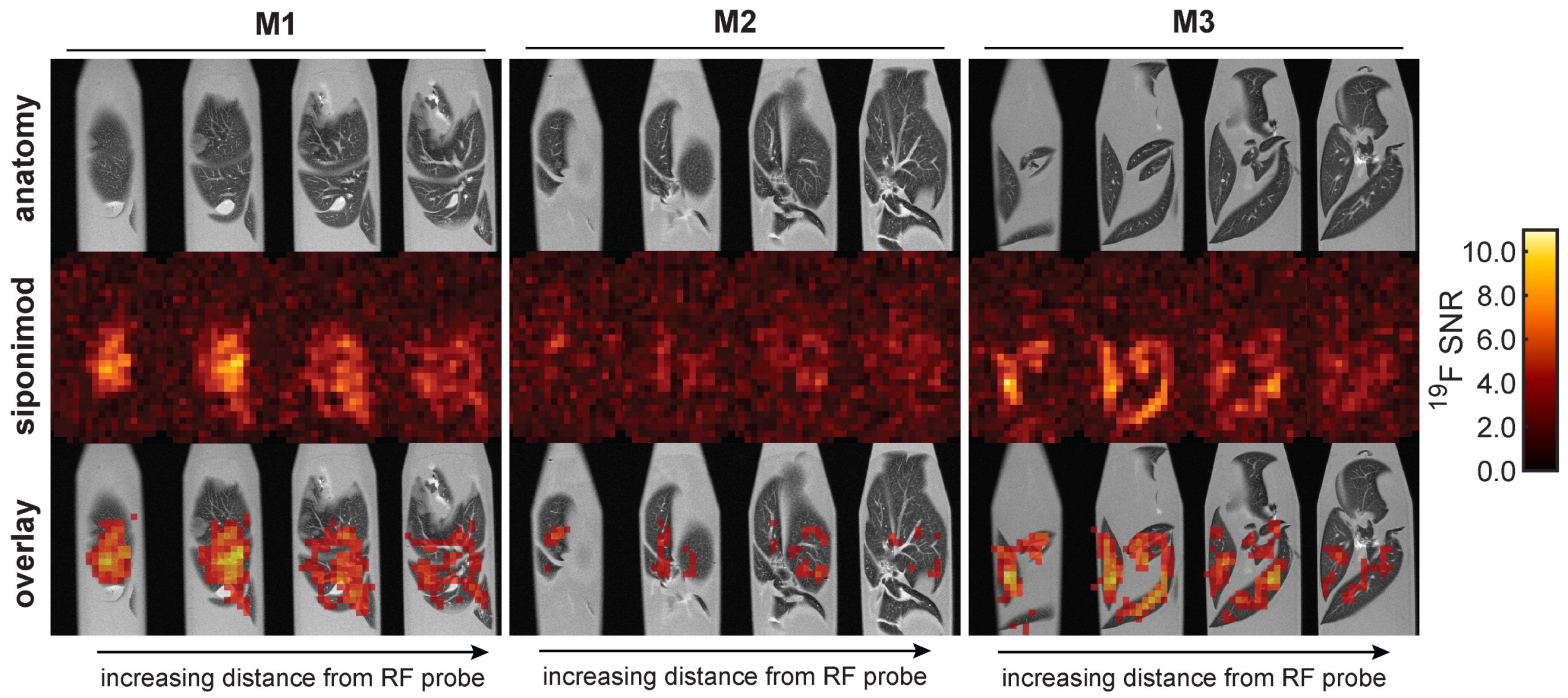
3D UTE ^19^F MRI in *ex vivo* liver tissues with 16 h acquisition time and 0.875 mm isotropic spatial resolution.

**Figure 7:**
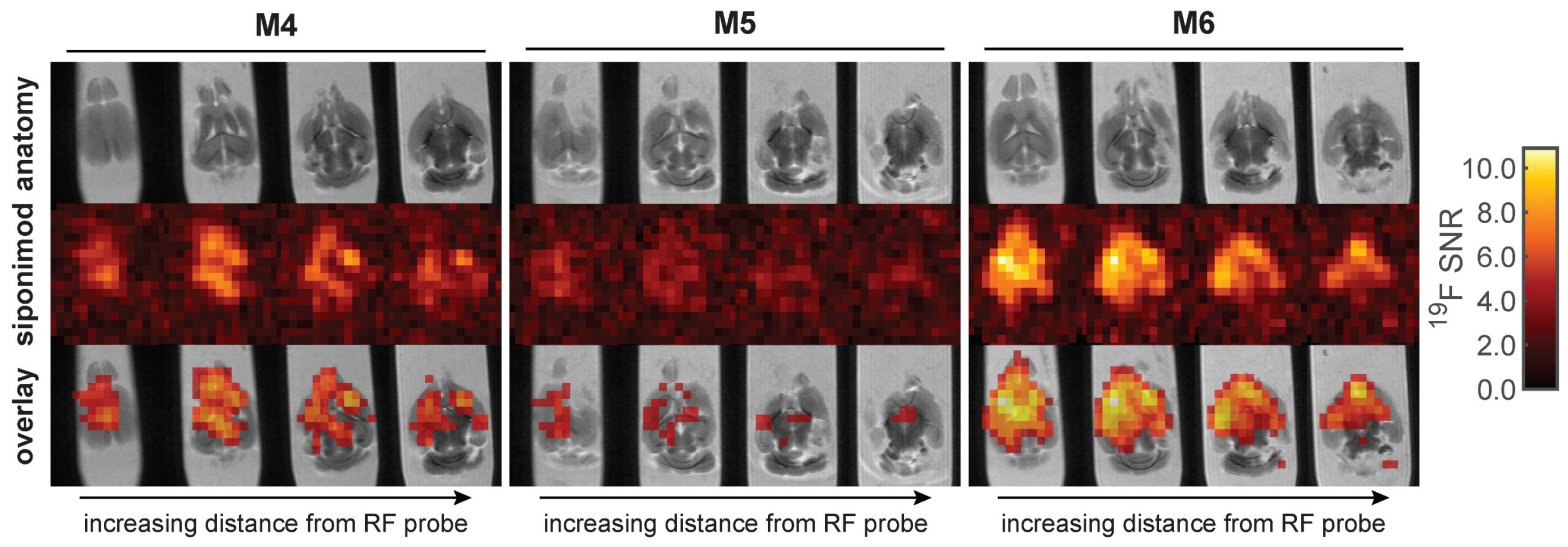
** 3D UTE ^19^F MRI in *ex vivo* brain tissues with 64 h acquisition time and 1 mm isotropic spatial resolution.** This protocol is 3.1 times more sensitive than that employed for the liver (Figure 6). Thus, equal SNR values correspond to 3.1 times lower concentrations of siponimod.

*Ex vivo* ^19^F MR images were acquired in liver (Figure 6) and brain (Figure 7) samples using the 3D-UTE MRI method. The ^19^F signal in the livers clearly follows the anatomy of the liver lobes, especially for mouse M3. Different anatomic segments showed different ^19^F signal intensities derived from siponimod. For example, less signal was detected in the upper liver lobe of mouse M1, compared to the lower lobe. Siponimod signals, especially those in the upper lobe of M1 were confined within the vascular boundaries. As expected, observed signal levels dropped with increasing distance from the RF coil. The overall much lower SNR in the liver ^19^F MRI signal of M2 (Figure 6) is consistent with the ^19^F MRS signal (Figure 5A).

Similar observations were made in the brain (Figure 7). The brain with the highest ^19^F MRS signal (M6) among all analyzed samples also showed the highest SNR in the ^19^F UTE MR images. Conversely, the brain with the lowest SNR in ^19^F MRI (M5) also had the lowest ^19^F MRS signal (Figure 5). Differences in siponimod-derived ^19^F signals could be resolved between white and grey matter, especially in the M6 brain (Figure 7). M4 and M6 show a clear difference between the cerebral and cerebellar regions of the brain. In the brain of M4, a region of low or no siponimod accumulation was observed within and around the right lateral ventricle. In a control measurement of a spiked serum phantom, the protocol employed for the brain resulted in 3.1-fold higher sensitivity than the protocol used for imaging the liver (Figure 6). Thus, based on similar recorded SNRs, we conclude that siponimod concentrations in the liver were about 3 times higher (Figure 6 and 7).

### Short acquisition time imaging of siponimod in the brain

**Figure 8:**
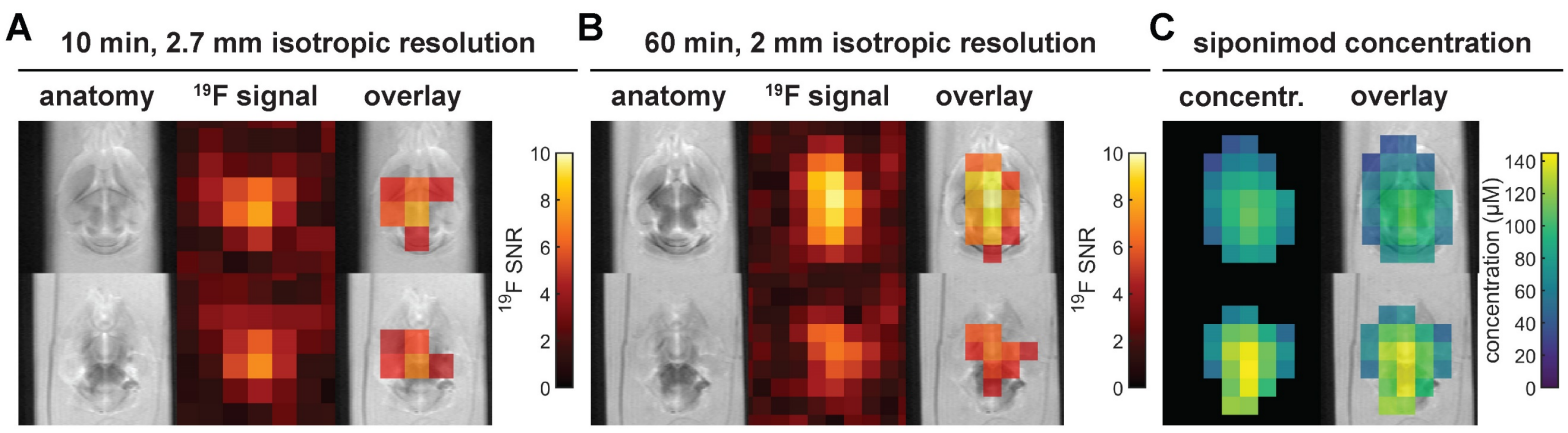
** Short acquisition time 3D UTE ^19^F MRI in *ex vivo* brain tissue and siponimod concentration estimation.** All subfigures show data acquired in the brain sample of mouse M6. (A) With 2.7 mm isotropic resolution, siponimod can be localized in only 10 min of acquisition time. (B) 60 min of acquisition enable imaging of siponimod at 2 mm isotropic resolution. (C) Siponimod concentration estimates computed by comparison of ^19^F SIs measured in a phantom with spiked serum and in the mouse brain. 4 acquisitions of the protocol shown in B were averaged and the same slices as in B are presented.

*In vivo* imaging of siponimod in the brain necessitates a substantial reduction of the acquisition time compared to the protocol above. Figure 8A and B present *ex vivo* images acquired at 2.7 mm or 2 mm isotropic spatial resolution, acquired in 10 min or 60 min, respectively. In both cases, siponimod could be localized within the brain and, particularly with 2 mm spatial resolution, a non-homogeneous SI distribution was also observed (Figure 8B). Comparison with SIs measured in a spiked serum phantom enabled an estimation of siponimod concentrations as well as an RF field (B_1_) inhomogeneity correction (Figure 8C). Drug levels up to 145 μM were reached in the brain of mouse M6.

To evaluate the possibility of imaging siponimod in a clinical setting, we determined the hardware sensitivity ratio between imaging a mouse brain at 9.4 T and imaging a human brain at 3.0 T at equivalent relative resolution. Due to the very close gyromagnetic ratios (≈6% deviation), this comparison could be performed at the ^1^H resonance frequencies. For the specific hardware investigated, we found that the larger voxel size viable for human brain measurements and higher RF channel count render the clinical acquisition 2.75 times more sensitive.

## Discussion

In the present study, we achieved for the first time *in vivo* and *ex vivo* ^19^F MR images of a fluorinated drug with disease modifying properties that is indicated in SPMS, a progressive form of MS with increasing brain degeneration.

In SPMS patients, the daily maintenance dose of siponimod is 2 mg. In an early phase 2 clinical trial in RRMS, one cohort received 10 mg daily up to 24 months 64. In a later phase 3 clinical trial in SPMS patients, a dose of 2 mg was used 65. A daily dose of 2 mg and 10 mg siponimod in humans corresponds to 0.03 and 0.17 mg/kg, respectively, for an average weight of 60 kg. In preclinical studies, doses ranged between 0.3 to 25 mg/kg, and therapeutic efficacy depended on species, application route, experimental design, as well as variations on the MS animal model, experimental autoimmune encephalomyelitis (EAE) 66-69. In these preclinical studies, siponimod was administered daily over extended periods (up to 4 weeks). A recent study reported a protective effect of siponimod, that was most pronounced at 15.5 mg/kg 66. The absolute oral bioavailability of siponimod is ≈84% in humans 70, 50% in rats and 71% in monkeys 71. The time (T_max_) for siponimod to reach maximum plasma concentrations (C_max_) after oral administration is 3-4 hours in healthy subjects 72. Steady-state C_max_ are reached after ≈6 days of once-daily dosing 73 and are 2-3 fold greater than after the initial dose 72, 73. In this proof-of-concept study we administered 4 mg (≈133 mg/kg) as a single, one-time application in healthy mice.

A dose-proportionality in steady-state siponimod levels in the brain was recently reported 69. Following repeated daily application (25 mg/kg) in female C57BL/6 EAE mice (over 27 days), concentrations of ≈45 μM were measured in whole brain homogenates 69. Considering this, a repeated application of 133 mg/kg (as applied in the present study but as a single dose) would result in brain concentrations of ≈240 µM. This is 2-3x greater than what we measured with fluorine MRI across different regions in the mouse brain following one application of siponimod, and is consistent with the expected 2-3-fold increase in C_max_ after several doses (steady-state after ≈6 days), when compared to one dose 72, 73.

Siponimod demonstrated very short transverse relaxation times in the presence of serum, analogous to our previous observations with teriflunomide, another MS drug 19, 74. We used serum as a biological medium to simulate the *in vivo* environment for siponimod. These investigations ultimately made the first ^19^F MR images of siponimod possible because we adapted acquisition protocols to the MR properties of the drug within the specific biological environment.

Siponimod is known to have a very high plasma protein binding capacity (>99.9% bound fraction) 75. The CF_3_ group in siponimod greatly improves its affinity to its target 76 as well as its lipophilicity, and thus likely its penetration into the CNS. A recent study in non-human primates using ^123^I- radiolabeling for *in vivo* single-photon emission CT showed penetration of siponimod into the CNS, with higher uptake in white matter versus grey matter 69. Therefore, the high plasma protein binding of siponimod does not appear to hinder its penetration into the CNS. This has also been described for many other drugs, which show high plasma protein binding, but which are nevertheless detected in the CNS in higher amounts than their unbound fraction in the plasma 77. Drug plasma protein binding reduces passive diffusion and uptake by peripheral tissue, as well as penetration into the CNS 78. However, apart from diffusion, active mechanisms such as receptor-mediated transport exist for drug uptake into the CNS 79. Siponimod targets both S_1_P_1_ and S_1_P_5_ receptors on BBB endothelial cells 80, which might contribute to an active transport into the brain. Inhibition of S1P1 receptors, specifically in endothelial cells, results in a transient opening of the BBB 81. Binding of smaller molecules such as siponimod to a much larger protein molecule is expected to strongly influence the MR resonance frequency (chemical shift) and relaxation rates 82, 83. While the observed change in chemical shift between solution in DMSO and in serum is moderate (≈ -2 ppm), the relaxation times were substantially shortened in serum. Similarly shortened relaxation times were observed in *ex vivo* tissue, and we expect that the properties in *in vivo* tissues will not differ substantially. The effect of protein binding on the transverse relaxation times (reduction by up to 98%) creates challenges for pulse sequences which are employed in the more common ^19^F MR methods such as point resolved spectroscopy (PRESS) or highly accelerated RARE in the case of perfluorocarbon imaging 37, 84-86.

Here, we determined radially encoded UTE to be the current best suited acquisition method for detecting siponimod under physiological conditions. In the context of ^19^F MRI, UTE acquisitions are mostly used for imaging fluorinated gases in the lungs 87, 88. Besides superior sensitivity for short T_2_^*^ compounds compared to RARE or bSSFP 42, UTE acquisitions offer intrinsic robustness to motion artifacts 89. The lower flip angles used with UTE reduce RF power deposition compared to RARE, which is essential for enabling efficient acquisitions in a clinical context 90. As shown in Figure 3A, UTE also offers a crucial enlargement of the sensitive FOV for acquisitions with transceive surface RF arrays, as was used in this study. We employed 2D multislice protocols for acquisitions with slice thicknesses of multiple millimeters, where slice encoding gradients do not entail substantial acquisition delays. For higher through-plane resolutions, 3D protocols were preferred. We chose tailored frequency encoding bandwidth to ensure that the signal readout did not extend beyond the signal decay. Short duration RF excitation pulses were chosen to achieve the lowest possible TEs. Unfortunately, this prohibits the use of adiabatic pulses which would improve transmission field (B_1_^+^) uniformity. More research is necessary to identify short RF pulses that balance a narrow frequency range with good penetration. Additionally, the readout trajectories could be further optimized for sensitivity and sharpness, for example by the implementation of spiral 91, twisted projection imaging 92, or density adaptation techniques 93, which were developed in the context of sodium (^23^Na) MRI 94, 95.

Using radially encoded 2D UTE ^19^F MRI in healthy mice *in vivo*, we localized siponimod in the stomach and the liver. Siponimod was also detected in the kidneys, brain, and thymus, even only 2.5-4.5 hours following a single oral dose. Nevertheless, differences in siponimod levels were observed among different tissues. Changes in the chemical environment e.g., pH could also influence the MR signal. Previously we showed that the ^19^F signal measured using MR spectroscopy with full relaxation was reduced in an acidic environment, as in the stomach 19. Therefore, acidosis which is prevalent in conditions associated with hypoxia such as inflammation or in tumors, would be expected to result in a decreased ^19^F signal.

*Ex vivo* ^19^F MR images of liver and brain samples revealed a non-homogenous distribution of the drug, contrary to our expectations from previous studies 96. In accordance with a recent report using *in vivo* SPECT-imaging of the siponimod analog [^123^I]MS565 in non-human primates 69, we observed a pattern of higher siponimod concentrations in white than in gray matter. We also observed signal variations in different anatomical regions, e.g., between the cerebral versus cerebellar regions of the brain, and between the liver lobes. No major differences were seen between brain cerebral and cerebellar regions in the previous SPECT study, even following the first 2 hours of intravenous administration 69. One important difference between the studies is that here we investigated the unmanipulated drug, identical to the form that is administered to SPMS patients as an oral dosage, whereas the SPECT study used a radiolabeled drug. The fluorine that we exploit for imaging with ^19^F MRI is intrinsic to the chemical structure of siponimod. The close match of tissue borders, e.g., in the liver lobes, between the anatomical images and the overlaid siponimod ^19^F MR images indicates an accurate localization of the drug. It is crucial that future studies investigate the signal distribution over repeated administrations of the drug, and importantly, during disease. This can be done using the EAE animal model. Different variants of the EAE model reproduce features of RRMS and SPMS 97, 98, and using ^19^F MRI to monitor siponimod distribution in conjunction with clinical disease activity could yield crucial insights. Our ^19^F MR spectroscopy results also indicate that the concentration of siponimod in the liver and the kidney are higher than those in the brain. Future studies will investigate the concentrations of siponimod in other organs apart from the central nervous system, also during pathology.

Ultimately, detection of fluorinated drugs with ^19^F MRI depends on the levels of the drug reaching the tissue, which depends not only on the administered dose but also on the drug distribution, e.g., in the CNS. Yet the general strategy of the current study - careful characterization of the MR properties of a fluorinated drug, and optimizing the MR methods to image *in vivo* and *ex vivo* - can in principle be extended to other fluorinated compounds relevant for diseases beyond MS. The sensitivity improvement offered by the CRP was crucial for achieving the detection limits necessary to detect a DMD in the mouse. Cryogenically-cooled surface RF probes can be employed for all preclinical studies which do not require whole-body coverage 36 and could enable 3D *in vivo* imaging of drugs which have so far only been investigated with spectroscopic ^19^F MR techniques or localized with very low spatial resolution 2D projection images, e.g., the cytostatic agents 5-fluorocytosine (5FC) including its metabolites 31, 99 and gemcitabine 100, the neuroleptic fluphenazine 101, the anti-depressants fluvoxamine and fluoxetine 33, 102, and the anti-fungal drug voriconazole 103. Our proposed spectral post-processing method could be used for the removal of all consistent, hardware-derived artifact signals and could also easily be adapted for dealing with confounding signals from anesthetics such as isoflurane. The suitability of UTE imaging protocols depends on the MR properties of the specific compound. Relaxation times under physiological conditions, especially T_2_^*^, have not been reported for most investigated fluorinated drugs. However, protein binding can be expected to have similar effects on other drugs 82, 83, and we also observed a binding-induced severe shortening of transverse relaxation times in both teriflunomide and teriflunomide derivatives 19, 74. We can assume that for compounds with short T_2_ and T_2_^*^, UTE will provide higher SNR efficiency than the often-employed magnetic resonance spectroscopic imaging (MRSI) protocols, due to the shorter TEs enabled by the absence of phase encoding gradients and the inherent oversampling of the k-space center. Fluorinated drugs with complex, multi-peak spectra or prominent metabolites present a challenge for standard UTE techniques, as chemical shift artifacts will lead to signal blurring and the downstream compounds cannot be distinguished. Especially at high magnetic field strengths and with large chemical shifts, this can be overcome with selective excitation of individual resonances. However, new techniques will need to be developed to overcome the resulting efficiency penalty and enable true multi-color ^19^F MRI, as has been demonstrated for perfluorocarbons 104.

*In vivo* imaging of siponimod in the brain at 1 mm isotropic spatial resolution will admittedly not be feasible in reasonable measurement times given the equipment and protocols described in this study. However, our exploration of lower spatial resolution, short acquisition time protocols suggests that informative mapping of the siponimod distribution *in vivo* in the mouse brain can be achieved, even as part of comprehensive multi-contrast or longitudinal study designs. We also found that, when considering differences in magnetic field strength, RF hardware, and species anatomy, detection limits are expected to be about 2.75 times lower in clinical measurements at equivalent relative spatial resolution (14 times larger voxel edge lengths) compared to preclinical imaging. Due to the availability of state-of-the-art hardware, the comparison was performed at the ^1^H resonance frequency leveraging the closeness of the ^1^H and ^19^F nuclei's gyromagnetic ratios (≈ 6% deviation). No substantial difference in transmission and reception RF field (B_1_^+^/B_1_^-^) penetration is expected between ^1^H and direct ^19^F measurements. The signal per nucleus is proportional to the third power of the gyromagnetic ratio (γ^3^) which impacts the measurement at 9.4 T and 3.0 T equally and thus does not affect the determined ratio 105. Crucially, choosing the ^1^H alternative for the comparison allowed the use of modern RF arrays with a geometry optimized for head imaging and excluded signal attenuation effects from operating the RF array of the clinical scanner outside the optimal bandwidth. While RF arrays developed for ^1^H imaging could be adapted for the construction of ^19^F coil designs, dedicated hardware with components free of fluorinated contaminants is necessary to achieve the best performance 90*.* The determined SNR improvement equates to 7.6 times faster acquisitions with equal sensitivity, which could be further enhanced by increasing the magnetic field strength (B_0_) of the clinical scanner from 3.0 T to 7.0 T (first systems are already in clinical use) or 10.5 T and beyond (first research systems available) 106-108. In practice, the sensitivity ratio will also depend on B_0_ effects on the relaxation times, primarily T_1_ relaxation. An increase of T_1_ with increasing B_0_, favoring high sensitivity at lower magnetic field strengths, is well documented for the ^1^H resonances of water and metabolites 109, but the opposite has been shown for the ^19^F resonance of perfluorocarbons 110, 111. Further developments of MR hardware, acquisition methods, and data processing are still necessary to shorten acquisition times, but the current study lays the groundwork for future, more elaborate studies, with the ultimate objective to translate ^19^F MRI into routine clinical practice.

Besides the trend towards higher magnetic field strengths 111, new developments in post-processing methods offer further possibilities to enhance ^19^F MRI sensitivity. The encoding trajectories commonly employed in UTE sequences are well-suited for undersampling and compressed sensing (CS) image reconstruction 112-114. Multiple publications have demonstrated that the increased averaging enabled by CS can lead to sensitivity improvements for low SNR MRI 86, 115, 116. The distribution of siponimod is less sparse than that of inflammatory lesions 86. At the spatial resolution achieved in this study, the signal distribution has important features at the scale of single voxels, and thus does not possess the sparsity necessary for successful application of conventional CS techniques. There is a need to investigate whether tailored reconstruction methods could overcome this challenge, and to determine whether CS can only be harnessed at enhanced spatial resolutions. Another post-processing method relevant to ^19^F MRI of fluorinated drugs is B_1_ correction for data acquired with transceive surface RF probes. This would allow the computation of quantitative concentration maps with the help of an external standard. Retrospective techniques based on the steady-state signal equation can be employed for the UTE sequence employed in this study 117, while more complex, model-based approaches would be recommended if a RARE sequence were employed 118. Finally, should sensitive hardware that enables alternating or even simultaneous ^1^H and ^19^F acquisition be available, motion correction techniques could be employed to reduce the need for restraining the subject under investigation and promote the successful outcome of the experiment without compromising animal welfare or patient comfort 119, 120.

Our goal is to harness the potential of ^19^F MRI as a powerful theranostic tool in the treatment and management of neurological pathologies such as MS. The stability of the siponimod signal in the stomach indicates a reduced gastric emptying and absorption of the drug. Future studies will investigate drug distribution in the absence of the confounding effects of anesthesia, since drug pharmacokinetics including absorption and distribution are likely to be affected 121. Nevertheless, the increasing signals observed in the liver during the experiment, and presence of siponimod in subsequently harvested tissues indicate that siponimod was indeed absorbed despite reduced gastric motility.

A study with radioactively labeled siponimod reported detection of metabolites in blood samples from healthy human volunteers 122. In mice, a cholesterol ester of siponimod, C_56_H_80_N_2_O_3_F_3_, still preserving the CF_3_ group, was reported as the main metabolite. This chemical change is unlikely to lead to significant differences in the chemical shift relative to the parent compound 122. In our study, *in vivo* and *ex vivo* MR spectroscopy did not reveal secondary peaks, except for the brain sample of mouse 1 (M1), which showed a small peak at -76.2 ppm. An increase in negative chemical shift (-60 to -80 ppm) indicates increased shielding of the CF_3_ group, which can occur as a result of branching or hydrogen bond donors in close proximity to the CF_3_ group 123, as is the case with a known hydroxylated siponimod glucoronide metabolite 122. However, considering that the -76.2 ppm peak is 0.6-1.1 ppm downstream of isoflurane 124, which sometimes contaminates the bore of the animal scanner following previous *in vivo* experiments, we cannot exclude isoflurane as a possible contaminant. Future experiments will involve repeated oral administrations of siponimod to determine whether the second -76.2 ppm peak will appear in the brain and other organs, especially in the context of neuroinflammatory pathology. It would also be appealing to determine whether siponimod metabolites can be differentiated from the parent drug in MS patients with *in vivo* ^19^F MRS. Alongside ^19^F MRI to image and localize drugs *in vivo*, localized MRS methods suitable for short T_2_ compounds, such as image-selected *in vivo* spectroscopy (ISIS), would be highly advantageous for non-invasive metabolite quantification to provide more insight into drug metabolism *in vivo* 125, especially during pathology.

## Conclusions

We imaged siponimod for the first time in its unmanipulated form, as administered to MS patients, using non-invasive ^19^F MRI. Further technological progress will enable the necessary accelerations required to provide measurement protocols for drug imaging in the CNS *in vivo*. While larger studies are necessary to draw conclusions on the pharmacokinetics of siponimod, the present study reports its distribution in the CNS and other organs. The methods used in this study will provide the groundwork for future clinical studies in MS patients to monitor drugs *in vivo*. These developments will ultimately enable the use of ^19^F MRI, which has thus far been an underexploited and underexplored tool in theranostics.

## Figures and Tables

**Table 1:** Pulse sequence parameters used for SNR comparison in the serum phantom (Figure 3).

		Repetition time, TR (ms)	Echo time, TE (ms)	Excitation flip angle α (°)	Echo train length, ETL
RT	RARE	350	1.5	90	8
	FLASH	10	0.89	15.2	-
	bSSFP	1,4	0.7	16.5	-
	UTE	10	0.14	15.2	-
PT	RARE	269	1.5	90	16
	FLASH	10	0.89	14.2	-
	bSSFP	1.4	0.7	26.3	-
	UTE	10	0.14	14.2	-

**Table 2:** ^19^F MR characterization of siponimod. T_2_^(bi)^ and T_2_^*(bi)^ are apparent relaxation times computed from a bi-exponential fit. T_2_^(mono)^ and T_2_^*(mono)^ denote the results of a mono-exponential fit. P values report a likelihood ratio test if T_2_ or T_2_^*^ decays follow a mono-exponential. β_T2_ and β_T2*_ give the ratio between the ^(a)^ and ^(b)^ components in the bi-exponential fits.

	DMSO, RT	DMSO, PT	Serum, RT	Serum, PT	*Ex vivo*
chem. shift (ppm)	-57.08	-57.06	-59.13	-59.06	-59.09
T_1_ (ms)	553.2±4.3	802.4±7.2	282.1±1.8	321.7±2.1	272.9±3.8
T_2_^(bi)^ (ms)	380.8±2.9	611.6±1.6	5.3±0.4	14.7±0.4	20.8±2.8
T_2_^(mono)^ (ms)	385.6±0.9	621.4±1.3	6.4±0.2	17.8±0.3	47.2±2.9
T_2_^(a)^ (ms)	226.0±32.4	205.3±39.9	4.7±0.6	8.0±0.9	9.4±1.6
T_2_^(b)^ (ms)	409.0±7.7	636.9±3.9	12.9±3.2	21.7±0.7	80.6±9.3
β_T2_	0.137±0.056	0.047±0.009	0.880±0.072	0.405±0.037	0.618±0.030
P_T2_	≪.001	≪.001	≪.001	≪.001	≪.001
T_2_^*(bi)^ (ms)	2.304±0.008	1.410±0.000	0.952±0.014	0.643±0.007	0.493±0.146
T_2_^*(mono)^ (ms)	3.710±0.018	2.040±0.018	1.316±0.012	1.696±0.012	0.551±0.017
T_2_^*(a)^ (ms)	1.037±0.006	0.778±0.004	0.336±0.018	0.325±0.004	0.240±0.196
T_2_^*(b)^ (ms)	6.12±0.02	3.94±0.02	1.83±0.04	3.42±0.03	0.65±0.11
β_T2*_ (1/ms)	0.554±0.002	0.629±0.002	0.425±0.014	0.671±0.003	0.260±0.286
P_T2*_	≪.001	≪.001	≪.001	≪.001	0.086

**Table 3:** SNR and SNR efficiency results for the MR pulse sequence comparison shown in Figure 3. Uncertainties were estimated as described in Starke et al. 60 with 270 pixel signal ROIs and 4100 pixel (UTE) or 6144 pixel (all other sequences) noise ROIs.

		RARE	FLASH	bSSFP	UTE
RT	SNR	8.5±0.1	3.48±0.07	2.44±0.06	13.0±0.2
	SNR_eff_ (1/√h)	4.24±0.05	1.74±0.03	1.22±0.03	6.50±0.08
PT	SNR	10.7±0.1	2.94±0.07	2.53±0.07	12.3±0.1
	SNR_eff_ (1/√h)	5.32±0.06	1.47±0.03	1.26±0.03	6.14±0.07
RT, CRP	pSNR	17.7	9.0	8.7	26.7
	pSNR_eff_ (1/√h)	30.5	15.6	15.0	46.2

**Table 4:** Peak SNR (pSNR) and area-to-noise ratio (ANR) in the MR spectra of *ex vivo* tissue samples (Figure 5).

	pSNR				ANR			
	liver	kidney	brain	thymus	liver	kidney	brain	thymus
M1	159.4	86.8	19.4	-	446.2	260.0	77.9	-
M2	88.5	47.2	12.5	-	223.5	135.4	49.3	-
M3	197.5	61.9	37.4	-	487.3	185.7	144.1	-
M4	489.5	166.0	36.6	22.6	927.6	398.7	129.2	44.0
M5	292.0	121.8	20.4	13.7	606.2	286.5	61.6	25.9
M6	462.5	128.4	53.0	15.5	953.5	340.6	193.2	36.0
mean	281.5	102.0	29.9	17.3	607.4	267.8	109.2	35.3
std. dev.	164.5	44.8	15.1	4.7	286.4	97.1	55.7	9.1

## Abbreviations

ANR: area-to-noise-ratio
a. u.: arbitrary units
BBB: blood brain barrier
bSSFP: balanced steady-state free precession
B_0_: static magnetic field
B_1_: radiofrequency field
CMC: carboxymethylcellulose
CNS: central nervous system
CPMG: Carr-Purcell-Meiboom-Gill
CRP: cryoprobe
DMD: disease-modifying drug
DMSO: dimethylsulfoxide
EAE: experimental autoimmune encephalomyelitis
FID: free induction decay
FLASH: Fast Low-Angle Shot
FOV: field of view
FWHM: full-width half maximum
ID: inner diameter
IP: intraperitoneal
MR: magnetic resonance
MRI: magnetic resonance imaging
MRS: magnetic resonance spectroscopy
MS: multiple sclerosis
PFA: paraformaldehyde
PSF: point spread function
pSNR: peak signal-to-noise ratio
PT: physiological temperature
RF: radio-frequency
RARE: Rapid Acquisition with Relaxation Enhancement
RRMS: relapse-remitting multiple sclerosis
RT: room temperature
SI: signal intensity
SPECT: single-photon emission computed tomography
SPMS: secondary progressive multiple sclerosis
SNR: signal-to-noise ratio
TA: acquisition time
TE: echo time
TR: repetition time
T_1_: longitudinal relaxation time
T_2_: transverse relaxation time
T_2_^*^: effective transverse relaxation time
UTE: ultrashort echo time
ΔTE: echo spacing
γ: gyromagnetic ratio
